# A Machine Learning Workflow of Multiplexed Immunofluorescence Images to Interrogate Activator and Tolerogenic Profiles of Conventional Type 1 Dendritic Cells Infiltrating Melanomas of Disease-Free and Metastatic Patients

**DOI:** 10.1155/2022/9775736

**Published:** 2022-10-12

**Authors:** Saraí G. De León Rodríguez, Paúl Hernández Herrera, Cristina Aguilar Flores, Vadim Pérez Koldenkova, Adán Guerrero, Alejandra Mantilla, Ezequiel M. Fuentes-Pananá, Christopher Wood, Laura C. Bonifaz

**Affiliations:** ^1^UMAE Hospital de Especialidades, Centro Médico Nacional Siglo XXI, Instituto Mexicano del Seguro Social, Unidad de Investigación Médica en Inmunoquímica, Mexico City, Mexico; ^2^Posgrado en Ciencias Biológicas, Universidad Nacional Autónoma de México, Mexico City, Mexico; ^3^Laboratorio Nacional de Microscopía Avanzada, Universidad Nacional Autónoma de México, Cuernavaca, Morelos, Mexico; ^4^UMAE Hospital de Pediatría, Centro Médico Nacional Siglo XXI, Instituto Mexicano del Seguro Social, Unidad de Investigación Médica en Inmunología, Mexico City, Mexico; ^5^Laboratorio Nacional de Microscopía Avanzada-IMSS, División de Desarrollo de La Investigación, Centro Médico Nacional Siglo XXI, Instituto Mexicano del Seguro Social, Mexico City, Mexico; ^6^Servicio de Patología, Hospital de Oncología Centro Médico Nacional Siglo XXI, Instituto Mexicano del Seguro Social, Mexico City, Mexico; ^7^Unidad de Investigación en Virología y Cáncer, Hospital Infantil de México Federico Gómez, Mexico City, Mexico; ^8^Coordinación de Investigación en Salud, Centro Médico Nacional Siglo XXI, Instituto Mexicano del Seguro Social, Mexico City, Mexico

## Abstract

Melanoma is the deadliest form of skin cancer. Due to its high mutation rates, melanoma is a convenient model to study antitumor immune responses. Dendritic cells (DCs) play a key role in activating cytotoxic CD8^+^ T lymphocytes and directing them to kill tumor cells. Although there is evidence that DCs infiltrate melanomas, information about the profile of these cells, their activity states, and potential antitumor function remains unclear, particularly for conventional DCs type 1 (cDC1). Approaches to profiling tumor-infiltrating DCs are hindered by their diversity and the high number of signals that can affect their state of activation. Multiplexed immunofluorescence (mIF) allows the simultaneous analysis of multiple markers, but image-based analysis is time-consuming and often inconsistent among analysts. In this work, we evaluated several machine learning (ML) algorithms and established a workflow of nine-parameter image analysis that allowed us to study cDC1s in a reproducible and accessible manner. Using this workflow, we compared melanoma samples between disease-free and metastatic patients at diagnosis. We observed that cDC1s are more abundant in the tumor infiltrate of the former. Furthermore, cDC1s in disease-free patients exhibit an expression profile more congruent with an activator function: CD40^high^PD-L1^low^ CD86^+^IL-12^+^. Although disease-free patients were also enriched with CD40^−^PD-L1^+^ cDC1s, these cells were also more compatible with an activator phenotype. The opposite was true for metastatic patients at diagnosis who were enriched for cDC1s with a more tolerogenic phenotype (CD40^low^PD-L1^high^CD86^−^IL-12^−^IDO^+^). ML-based workflows like the one developed here can be used to analyze complex phenotypes of other immune cells and can be brought to laboratories with standard expertise and computer capacity.

## 1. Introduction

Cancer is one of the most complex diseases that humanity has faced. This has led to the search for more powerful approaches to understand its biology in greater detail. The analysis of the role of the immune system has gained prominence due to the control it exerts over the disease. Of particular importance is the identity and functionality of those cells that infiltrate the tumor microenvironment (TME) [1]. Melanoma is considered the human tumor with the highest number of genetic mutations, making it a highly immunogenic cancer [2]. Despite this, melanoma is one of the deadliest cancers. Even though melanoma is more common in Caucasian populations, it is one of the fastest-growing malignancies in Mexico, with an increased incidence of 500% in recent years [3, 4]. The World Health Organization (WHO) also reports an increase of 115% in mortality, highlighting the need to study melanoma in the Mexican population [5, 6].

Because of its tumor mutational burden, melanoma is an ideal model for in-depth studies of the immune composition of the TME and its relationship with prognosis [2]. Dendritic cells (DCs) are the cells in charge of shaping the response of CD8^+^ T lymphocytes, which have been extensively studied in melanoma and other cancers as key effectors of the antitumor response [7]. DCs are the most important antigen-presenting cells (APC), and, as such, DCs coordinate antigen presentation to CD4^+^ T or CD8^+^ T lymphocytes on Major Histocompatibility Complex (MHC) II or I, respectively [8]. Moreover, the resulting activity of the T cell depends on the capacity of the DC to provide costimulatory or coinhibitory signals, such as those mediated by cytokines, CD40, CD80/CD86, PD-L1, or the enzyme indolamine dioxygenase (IDO) [9]. Classical dendritic cells type 1 (cDC1s) are the least proteolytic of all APCs. This makes cDC1s very efficient at delivering antigens to the lymph nodes, having the greatest capacity to undergo MHCI antigen cross-presentation to efficiently activate CD8^+^ T cytotoxic responses [10–12]. cDC1s are distinguished by the expressions of CD11c, HLA-DR, and BDCA-3 [8, 9].

For many years, DCs have been considered to function as activators or inhibitors of the immune response in a mutually exclusive manner [13]. More recent studies support the notion of different states of activation of DCs based on the coexpression of activating or inhibitory molecules. These studies have challenged the concept of opposing roles for DCs as strictly immunogenic or tolerogenic [14, 15]. For instance, recent data support that there are specific cDC2s subtypes expressing activator and inhibitor molecules associated with an extended overall survival of patients with head and neck squamous cell carcinomas [16]. Less clear is whether there are also different subtypes of cDC1s, as well as their possible relationship with disease control. Transcriptomic studies confirm that a cDC1 signature is associated with improved patient survival in melanoma and other cancers [17, 18]. However, most of our knowledge comes from peripheral blood cells or transcriptomic studies, and we still lack information about the cDC1 subtypes present in the TME based on proteomics approaches [8, 19].

A comprehensive examination of the TME requires multiparametric approaches to assess the presence of specific immune cells, as well as markers of performance, particularly those associated with tumor control. Multiplexed immunofluorescence (mIF) involves the analysis of several markers within a single sample, providing information-rich images that report the presence of different immune cells coexisting in the TME, as well as their spatial relationship to each other and with tumor cells, ultimately providing a snapshot of the biological architecture of the tissue [20]. Manual analysis of a set of mIF images, in which each image may contain hundreds to thousands of cells with multiple markers, is a time-consuming and error-prone process that involves compromises, such as minimizing the number of regions analyzed, with the risk of introducing subjective biases in their selection [21].

Artificial intelligence (AI) is a field of data science focused on programming a machine to perform multiple tasks like a human. Machine learning (ML) is an application of AI in which algorithms read, analyze, and learn from input datasets to subsequently make informed decisions. Deep learning (DL) is a subset of ML techniques, whereby algorithms are structured into layers that form an artificial neural network, enabling computers to effectively represent data with multiple levels of abstraction to progressively extract higher level features [22]. DL has proven particularly powerful in clinical image analysis over recent years, for instance, in disease diagnosis [23]. There are several advantages to using DL over a manual image-based diagnostic, for instance, improved accuracy [24, 25], greatly improved throughput, lack of subjective bias, and increased reproducibility [21].

In mIF image analysis, it is critical to accurately discriminate individual cells and profile those cells according to the expression of specific markers. Several DL models have been developed to automatically segment nuclei [26–28], and ML algorithms to speed up the classification process. The combination of mIF and ML powerfully improves the accuracy, throughput, and rigor of the characterization of the TME [29, 30]. In this study, we were able to establish a workflow for the analysis of cDC1s in melanoma based on the evaluation of mIF images using DL and ML strategies. This workflow allowed us to identify and profile cDC1s in the TME and evaluate their phenotypes associated with the control of the disease. To the best of our knowledge, this study is the first that combines the use of mIF and AI to profile the expression of nine parameters of identity and performance of TME-infiltrating cDC1s in clinical samples. This workflow can be adapted to assess immune and nonimmune cells in a variety of tissue samples.

## 2. Materials and Methods

### 2.1. Samples and Images Acquisition

#### 2.1.1. Melanoma Samples

Of 36 samples available, we selected seventeen paraffin blocks from tissue resections of patients with diagnosed melanoma. These samples were obtained from the Pathology Department of the Hospital de Oncología, Centro Médico Nacional Siglo XXI, Mexico City, Mexico, with the approval of the scientific and ethics committees (protocol R-2019-785-05). In the study cohort, we included patients whose melanomas were metastatic at diagnosis (*n* = 6), along with samples derived from patients who did not have metastases and remained disease-free after two years of follow-up (*n* = 11). This sample selection strategy was chosen to have greater certainty of exploring both a TME infiltrated with cells related to disease control and cells present in a progressive melanoma (clinical data are depicted in Table 1). Of this group of patients, a representative group was selected for mIF staining and multiplexed imaging including five metastatic and five disease-free patients. Additionally, five resection products of skin were obtained from individuals without cancer and included as controls for nontumor skin.

#### 2.1.2. Immunofluorescence (IF) Staining

Tissue sections of 15 *μ*m were mounted on glass slides (Superfrost Plus Green). Slides were placed for 45 min into an oven (70°C) to remove excess paraffin. Tissues were rehydrated with a Xylol/ethanol train of solvents. Antigen retrieval was performed using citrate buffer pH 6.0 (sodium citrate 10 *μ*M) at 90°C for 20 min. Then, samples were permeabilized with a solution of 10 mg/mL bovine serum albumin, 5% horse serum, 0.02% sodium azide, and 0.3% Triton for 2 h. Following permeabilization, samples were incubated with different primary antibodies: rabbit anti-human CD11c (ab52632, Abcam), rat anti-human HLA-DR (YD1/63.4.10, Invitrogen), mouse anti-human BDCA-3 (ab6980, Abcam), rat anti-human CD40 (ab22469, Abcam), and rabbit anti-human IDO (ab122402, Abcam). Primary antibodies were revealed with secondary conjugated antibodies: anti-rabbit Alexa Fluor 488 (711-547-003, Jackson ImmunoResearch), anti-rat Alexa Fluor 594 (712-585-153, Jackson ImmunoResearch), and anti-mouse Alexa Fluor 647 (715-605-151, Jackson ImmunoResearch). After that, nuclei were stained with Hoechst (Invitrogen) for 10 min. Sections were mounted with 10% glycerol in PBS. Images were acquired after this step of staining and used for analysis. To perform the second round of staining, coverslips were removed by soaking the slides in 1X PBS, and samples were processed as described below.

#### 2.1.3. Multiplexed Immunofluorescence (mIF)

mIF is based on a technique of tissue cyclic immunofluorescence (t-CyCIF) [11]. After the first step of immune labeling described above, tissues were incubated with a solution containing 2% hydrogen peroxide and 4.5 *μ*M sodium hydroxide at the presence of white light for 1 h, according to the original protocol, followed by 10 min of UV light irradiation to remove any signal that could remain from the first step of staining. Then, slides were incubated overnight with anti-human antibodies: FITC-conjugatedanti-CD40 (555588, BD Biosciences) or anti-CD11c (301604, BioLegend), APC-conjugatedanti-PDL1 (329708, BioLegend) or anti-IL-12 (p40/p70) (554576, BD Biosciences), PE-conjugated anti-HLA-DR (307606, BioLegend), anti-CD86 (305438, BioLegend), or APC-anti HMB45. Nuclei were stained with Hoechst (Invitrogen) for 10 min. Images were acquired from the same field after each staining step (see Table 2). Patients selected for each staining are described in each figure.

#### 2.1.4. Confocal Microscopy

Micrographs were obtained on a Nikon Ti Eclipse inverted confocal microscope (Nikon Corporation) using NIS Elements v.4.50. Imaging was performed using a 20x (dry, NA 0.8) objective lens. Additional magnification (3.4x) was attained through Nyquist's sampling during image acquisition. Three areas of high level of immune infiltrate from each group of patients and controls were taken to quantify the density of cDC1s. Images were preprocessed using FIJI ImageJ Software [31] to adjust the brightness and contrast, assign consistent look-up tables, and set the channels order prior to their alignment.

#### 2.1.5. Whole Slide Scanning

The slide scanner APERIO FL (Leica Biosystems) was used to obtain images of the complete melanoma specimen, with a 20x objective and defining the adequate time to exposure per channel with eSlide Scan Scope v12.3.3. Analysis of images was processed using FIJI software. To identify cDC1 in the whole slide scan, a mathematical treatment was given in order to amplify the signal.

#### 2.1.6. Channel Alignment

mIF images were obtained from the same samples labeled for two different sets of markers imaged on consecutive days. On the second day, the same fields of view were first localized by eye, and images were captured and then aligned using nuclei as reference on both staining steps. For this purpose, the ImageJ plugin descriptor-based registration (2d/3d) was employed [28]. To use this tool, we selected interactive brightness detections and a 2d rigid transformation model considering approximate prealignment. Image registration is achieved through correlated descriptors in nuclei-stain channels.

### 2.2. Cell Nuclei Segmentation and Training of Different Deep Learning Architectures

#### 2.2.1. Manual Annotation

There are several cell nuclei segmentation algorithms based on DL with pretrained models described in the literature (see below). The pretrained models have good performance when they are used to test images with similar characteristics (similar noise, nuclei shapes and sizes, intensity decay profiles, etc.) to those images used during training [33]. When this is not the case, erroneous results or artifacts can be generated [34, 35]. Some DL models can be more efficient at producing better results than others, and it is important to compare them. Hence, it is usually recommended to retrain the DL algorithms with manually annotated (ground-truth) images from the experimental model and a dataset of current interest to adjust the weights of the models and optimize the segmentation output.

In our case, we selected 12 representatives IF images from the total pool of 66 images available across the 3 categories of patients tested. We manually annotated the nuclei of each cell in these images, obtaining a total of 19,280 nuclei that constituted our own ground-truth image dataset which was used to train and compare different DL models. Manual annotation was carried out using the ImageJ plugin Annotater, as well as a tablet and a stylus pen to delineate and define each nucleus. The stylus pen is a more precise and sensitive tool than a computer mouse, and the tablet was synchronized with the computer interface using the SuperDisplay software.

#### 2.2.2. Deep Learning Models for Cell Nuclei Segmentation

The obtained images of nuclei (channel 1 using Hoechst) with their manual annotations (nuclei masks) were used to train and compare four different DL approaches for nuclei segmentation: U-Net3-class [36], Stardist [28], SplineDist [27], and Cellpose [26]. All these algorithms are based on the same underlying U-Net architecture [37], with the main difference between them being the output of the U-Net. Each algorithm has a methodology to identify individual nuclei from the output of the U-Net.

The U-Net architecture consists of two parts: the first encodes information by employing convolutional and downsampling layers, and the second decodes the information to the desired output by using convolution and upsampling layers. The output of the basic U-Net is a binary mask, where pixel values of 1 correspond to nuclei and pixel values of 0 correspond to background. The U-Net can generate good segmentation results; however, it cannot identify individual cells, as it merges overlapping or touching cells [37]. The U-Net3-class is an extension of the basic U-Net [36]. It returns two images as output, a binary mask with the nuclei segmented and a probability map corresponding to the boundary for each individual nucleus. This architecture was proposed to handle overlapping and touching cells [36].

Stardist is robust at detecting overlapping and touching cells, as well as generating a unique identifier for each nucleus, taking the prior knowledge that cells form a convex shape [28]. The Stardist network predicts *N* radial distances for pixels inside a nucleus to their nucleus boundary, as well as a probability map with high values assigned to pixels near to the cell nuclei center. The pixels with high cell nucleus probability are considered candidates to represent the center of a nucleus (the *N* radial distances are used to obtain its boundary). Because there can be more than one candidate per individual nucleus, Nonmaximum Suppression (NMS) is used to identify a single candidate per cell [34]. The limitations of Stardist include the requirement of a star-convex polygon representation for the object to be segmented (convex shape) and many radial distances (usually *N* = 32), which are used to generate a good approximation to the boundary of the object (relatively large objects may require extra radial distances).

SplineDist is an extension of Stardist which overcomes its main limitation of requiring the object to be convex [27]. SplineDist uses control points and spline models instead of radial distances. The output of the U-Net architecture in SplineDist is 2*∗N*+1 images which correspond to *N* angles, *N* distances associated with each angle, which are used to obtain *N* (two-dimensional) control points, and the cell nucleus probability. Similar to Stardist, SplineDist selects pixels with high nucleus probability as candidates for representing the nucleus boundary. NMS is used to identify a single pixel candidate per cell. The selected pixel for each nucleus allows obtaining *N* control points, which are used to delineate the cell nucleus boundary (splines-fit).

Cellpose is another approach that predicts three images corresponding to the horizontal and vertical gradient of a heat diffusion simulation (the heat diffusion simulation is computed only during training of the model) and the nucleus probability [26]. The nucleus probability corresponds to the output of the basic U-Net, that is, a binary mask corresponding to the segmented nuclei. The predicted gradients are used to postprocess the binary mask to assign (by gradient flow tracking) each pixel to a unique cell, hence segmenting individual cells.

#### 2.2.3. Training the Deep Learning Models

Training a DL architecture is usually a difficult task requiring programming skills (e.g., Python, TensorFlow, PyTorch, etc.) and access to high computational resources (a GPU with at least 12 GB GPU being recommended). ZeroCostDL4Mic is a toolbox providing Jupyter Notebooks to be used in the Google Colab environment for training DL models in the cloud [29]. The ZeroCostDL4Mic platforms are attractive as researchers no longer require programming skills to do the training nor local access to a powerful computer workstation.

ZeroCostDL4Mic includes Jupyter Notebooks, which are used to train various DL architectures and, most relevant for this study, to train and export Stardist, SplineDist, and Cellpose models. Although Google Colab allows free GPU access (usually 12 GB), it limits such sessions to 12 h, and the session can be prematurely finished if there are periods of inactivity (idle time); hence, the training can be lost and must be reinitiated. Therefore, we modified the Jupyter Notebooks from ZeroCostDL4Mic to run them in a local environment (Nvidia GeForce RTX 2080 Ti GPU 11 GB). The U-Net (3-class) network was also run in the same local environment and accessed through the Jupyter Notebook from the GitHub repository [35].

Evaluating the performance of a DL algorithm requires three sets of images for training, validation, and testing. The training set allows training the DL algorithm from scratch by learning the weights of the deep learning model, such that the nucleus images in the training set are mapped to the corresponding manually annotated images. The validation set was used during the training of the model, to ensure that the mapping is accurate for images (of nuclei) which were not included in the training set. Finally, the test set was used to measure the performance of the model after completing the training for the task of nucleus segmentation. Each of these sets should contain pairs of images composed of a cell nucleus image and its corresponding manually annotated, ground-truth nuclei mask image. The 12 image pairs, representing a total of 19,280 cells, were distributed randomly between training (8 images), validation (2 images), and testing (2 images) sets. A training set with few images may have difficulties to create a suitable learning model. If this is the case, it is recommended to increase the number of training images by a process called data augmentation. Data augmentation consists of slight modifications to the original images to increase the diversity of the training set. These transformations include random image rotation, flips of the axes (*x*-axis and *y*-axis), and changes of the intensity values. Automated data augmentation was employed in the training of all models.

#### 2.2.4. Evaluation Metrics

We use standard metrics (Precision, Recall, Average Precision, and *F*1-Score) to measure the performance of the DL models for the task of nuclei segmentation. These metrics require counting the number of cells correctly detected by the DL model (True Positive (TP)), incorrectly detected (False Positive (FP)), and not detected (False Negative (FN)). TP corresponds to the number of manually delineated cells correctly identified by the DL model, FP are cells detected by the DL model but absent in manually annotated images, and FN are cells manually annotated but not identified by the DL model. The evaluation metric Intersection over Union (IoU) is used to count these values; it measures the area of overlap between the ground-truth and generated masks (segmentations) and has values in the interval [0, 1], where a value of zero indicates no overlap between the two masks and a value of 1 indicates that the two masks overlap perfectly. Given any individual nuclei mask predicted by a deep learning model (*I*_*P*_) and an individual mask in the manual annotation (*I*_GT_), IoU is defined as

(1) $$\begin{array}{c} \mathrm{IoU}=\frac{I_{P}\cap I_{GT}}{I_{P}\cup I_{GT}}. \end{array}$$

If there is an individual mask in the manual annotation, such that IoU is greater than a fixed threshold *T*, then the individual mask *I*_*P*_ is counted as correctly detected (TP); otherwise, it is counted as incorrectly detected (FP). A mask *I*_GT_ in the manual annotation is counted as FN if IoU is lower than *T* for all the individual masks predicted by the DL model (*I*_*P*_). The values TP, FP, and FN are used to compute the metrics Precision, Recall, Average Precision, and *F*1-Score as follows:

(2) $$\begin{array}{c} \text{Precision}=\frac{\mathrm{TP}}{\mathrm{TP}+\mathrm{FP}},\\ \text{Recall}=\frac{\mathrm{TP}}{\mathrm{TP}+\mathrm{FN}},\\ \text{Average Precision}=\frac{\mathrm{TP}}{\mathrm{TP}+\mathrm{FN}+\mathrm{FP}},\\ F1=2\frac{\text{Precision}\cdot \text{Recall}}{\text{Precision}+\text{Recall}}. \end{array}$$

Precision is a metric to measure the performance of the model at predicting, while Recall is a measure of the model at producing erroneous detection. Average Precision considers the three values (TP, FP, and FN). The metrics are normalized in the range [0, 1] with lower values indicating a bad performance. *F*1-Score is a measure that combines Precision and Recall, giving an overall measure of the performance of the model.

### 2.3. Marker Classification and Evaluation of the Deep Learning Models

For marker classification and identification of cDC1 cells, two open-source software programs were tested, Annotater and QuPath, using the IF images that correspond to the 12 manually annotated nucleus images employed to generate the DL models. The IF images used for training were excluded from the final analysis, for which the remaining 54 images were used.

Annotater is an ImageJ plugin [26] to manually annotate objects of interest in the images. Additionally, given an image with the individual nuclei annotated (manually or by a ML/DL algorithm), it has the capability to identify if a nucleus is positive for a given marker. Annotater has three options to identify markers and assign them to individual nuclei: (i) manual annotation, where the user selects all nuclei in the image that are positive for a given marker; (ii) a thresholding approach, where a nucleus is identified as positive for a marker if a defined percentage of the pixels in or close to the nucleus have intensity value higher than a threshold; and (iii) ML annotation, for which the user identifies and selects a few nuclei positive for the markers (typically 5–10 nuclei) and a similar number of negative control nuclei (without marker), from which a logistical regression algorithm is trained to automatically classify the remaining nuclei into these two classes. The user has the option to revise the result of the model and refine the ML model and/or the results directly. The trained ML marker model is then applied to automatically classify cells in the remainder of the total image cohort. We employed the third modality in this study.

QuPath is an image analysis software designed as a user-friendly, extensible, open-source solution for digital pathology and whole slide image analysis [25]. Similar to Annotater, QuPath can generate ML models to classify cells according to their associated marker expression. The main difference to Annotater is having random tree, artificial neuronal networks, and k-nearest neighbor as the ML algorithms for classifications instead of logistic regression. Two scripts were implemented to execute measurements and import the binary mask from segmentation models. The training was performed based on their object classifier random trees algorithm and artificial neural network (a multilayer perceptron).

### 2.4. Statistical Analysis

All data are presented as the mean ± SD (standard deviation). The following comparisons were made with data derived from the immunofluorescence images: cell percentages from controls, MT, and DF patients. Statistical analyses were performed using One-Way ANOVA with multiparametric comparison. In case of mIF, comparisons were made between MT and DF groups applying Student's *t*-test. All statistical analyses were performed using Prisma Software (GraphPad 8). Statistical significance was defined as ^*∗*^*p* < 0.05,^*∗∗*^*p* < 0.01,^*∗∗∗*^*p* < 0.001,  and ^*∗∗∗∗*^*p* < 0.0001.

## 3. Results and Discussion

### 3.1. Establishment of an Optimized Workflow to Compare Different DL Algorithms

Although there is evidence in the literature about the presence of cDC1s in melanoma [17, 18], the expression of activation and inhibitory markers and their relationship with the evolution of the disease remain unclear. We compared several DL and ML algorithms to establish a useful methodology to analyze IF and mIF images to study the profile of TME-infiltrating cDC1s. To speed up image analysis and reduce possible subjective operator bias, we set out to explore the utility of generating DL models for cell segmentation and ML approaches to assist cell classification. We also evaluated the performance and friendliness of different DL and ML methodologies and platforms that have been made available to the research community.

**Figure 1:**
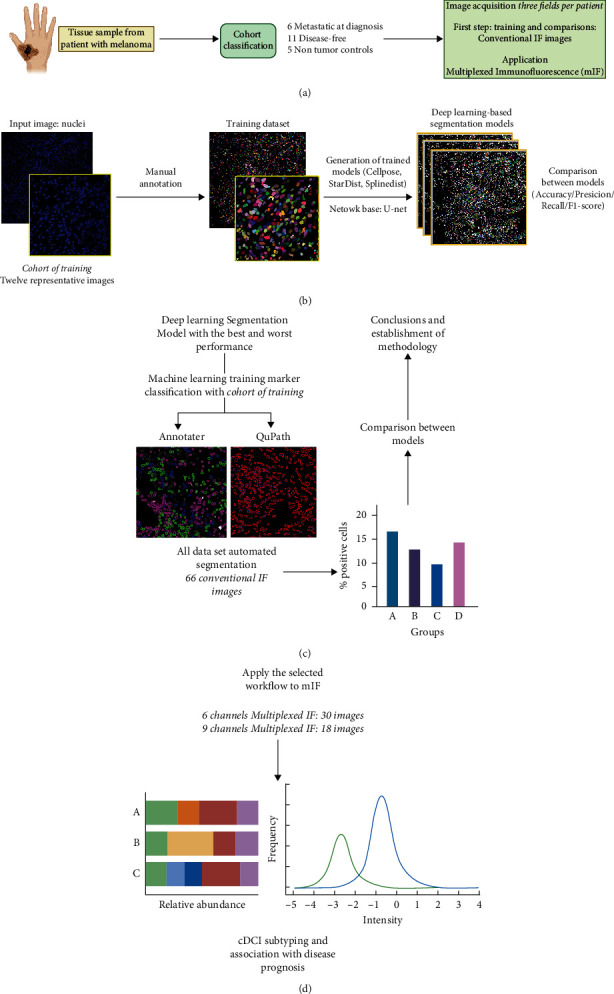
Overview of the workflow used to compare the performance of deep learning models and establish a pipeline to analyze the presence of cDC1s in immunofluorescence images of melanoma. (a) Schematic representation of the patient's cohort and description of all the conventional immunofluorescence (IF) and multiplexed IF (mIF) images analyzed. (b) Generation of the trained models for nuclei segmentation. Twelve random representative images with a total of 19,280 nuclei were used and divided in three sets: eight images were used during training, two for validation, and two for testing. Input images from the first set were annotated manually to obtain the training dataset (ground-truth). These data were used to train different Convolutional Neural Networks based on U-Net architecture and compare them to select the best workflow of analysis. (c) Comparison of Annotater and QuPath algorithms for training of marker classifiers. The training algorithms with the best and worst performance in nuclei segmentation were tested with the marker classifiers to evaluate the impact of selection in quantitative results and establish the best workflow. (d) With the established methodology, mIF images were used to evaluate the phenotype of melanoma-infiltrating cDC1s and correlate them with disease evolution.

To systematize this characterization, a workflow was established to evaluate and perform comparisons of the advantages and disadvantages of the most common algorithms. 17 patients diagnosed with melanoma were selected, 11 of whom were disease-free two years after diagnosis, and six already had evidence of metastases at diagnosis. Skin samples of five subjects with no evidence of cancer were also included as nontumor controls. An initial conventional IF staining of CD11c, HLA-DR, and BDCA-3 was used to identify cDC1 cells, with Hoechst staining used to define the position of nuclei (Figure 1(a)). With the IF images, two critical stages were established: segmentation of nuclei and the detection of positive and negative cells for the specific markers.

For segmentation of nuclei, we selected 12 representative images (of a total of 66) for manual annotation to establish the ground-truth dataset (summing a total of 19,280 nuclei). This ground-truth dataset was used to train, validate, and test models created on four separate DL architectures and to compare metrics of their performance (Figure 1(b)). For the second stage, we compared two software programs developed for annotation and training of marker classifiers, Annotater and QuPath, assessing which one of them performed better in identifying and assigning immune cell marker profiles to individual cells segmented by the DL models generated in the first stage (Figure 1(c)). Finally, with all the data from the different comparisons, the final workflow was established to analyze mIF images to obtain information about the density of cDC1s in the TME of melanoma (Figure 1(d)).

### 3.2. First Step: Generation of Our Own Deep Learning Models for Nuclei Segmentation

The most direct route to implementing DL-based segmentation in image analysis workflows is to use one of many pretrained models, some of which are available as modules within popular image analysis software, for example, Stardist default pretrained models implemented in ImageJ or QuPath. For this, the user's images are processed with minimal parameter adjustments for optimization; however, performance may not be optimal if the user's own images were obtained under conditions that differ from those used in the training image sets employed for the pretrained models. Higher performance requires generating DL models with training data derived from the user's own dataset. A wide variety of DL architectures are available to train nuclei segmentation models, from complex models with special computational requirements such as U-Net3-class to more accessible coding-free architectures on open-access high computing platforms, such as the already mentioned Stardist itself (based on the U-Net architecture).

To determine which of these models was the most suitable for our data, we first generated the “ground-truth” or the standard data to compare. For this, 12 representative images were selected from 66 images of metastatic, disease-free, and control groups, delineating a total of 19,280 nuclei to train the models (Figure 1(a)). In total, an average of 1600 nuclei per image were manually delimited over a total of 46 hours invested (Supplementary Table 1). Then, manually annotated images were used for training, validating, and testing DL models prior to the evaluation of their performance upon processing the complete image set (Figure 1(b)).

### 3.3. Parameter Setting for Training Deep Learning Algorithms for Nuclei Segmentation

The performance of the DL algorithms was optimized using several combinations of parameters as follows: (i) three patch sizes were tested, 256 × 256, 512 × 512, and 1024 × 1024 (original image size covering the whole sample); (ii) the number of iterations for training (epoch) was varied until the performance of the algorithm converged; (iii) data augmentation was set to 0, 2, 5, 10, and 40, where these numbers indicate the number of times the original training set is increased (e.g., 5 increased the training set to 40 images); (iv) the batch size was set as large as possible to occupy all the available GPU RAM memory; and (v) the learning rate was fixed to different values (0.1, 0.01, 0.001, 0.0003, 0.0002, 0.0001, and 0.00001).  Table 3 summarizes the parameters selected for each of the deep learning models. First, Stardist was trained using all the above-mentioned parameters. Data augmentation significantly increased the performance of the models, with 5 being a good performer by using the metric *F*1-Score. Since data augmentation comes at a significant cost in total training time, the other DL architectures were subsequently tested with data augmentation up to 5. SplineDist was the model requiring the greatest amount of GPU memory, particularly with the largest patch size. The time expended in training SplineDist was relatively high, such that the ZeroCostDL4Mic platform proved unsuitable for training SplineDist due to the time limits imposed for access to cloud computing on Google Colab.

### 3.4. Performance of Each Deep Learning Algorithm Tested

**Figure 2:**
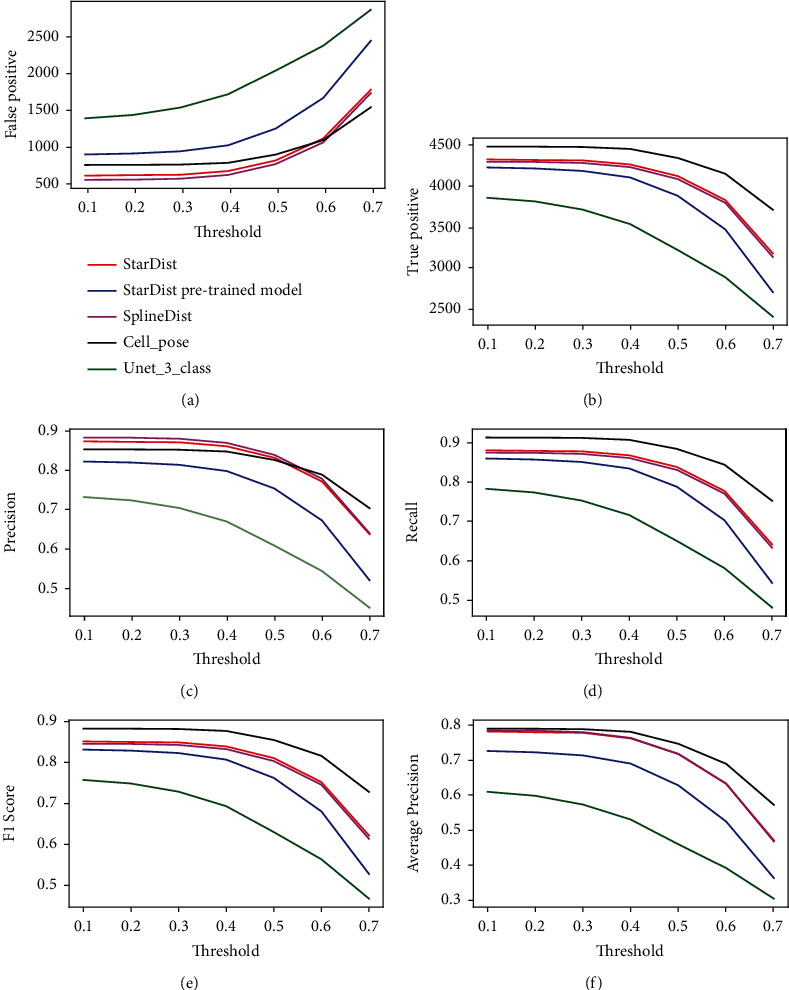
Comparing several deep learning models for nuclei segmentation. ((a)–(f)) Graphs depicting the performance of Stardist, Stardist pretrained model, SplineDist, Cellpose, and U-Net3-class on two test images using the metrics False Positive, True Positive, Precision, Recall, *F*1-Score, and Average Precision. The *x*-axis of each graph corresponds to different threshold values used to evaluate the metric IoU necessary to compute the metrics (see Section 2.2.4), with higher values corresponding to a higher overlap between the manual annotation and the predicted nuclei by the models. Lower values of False Positives (graph a) correspond to better models, while higher values of the other metrics correspond to better models.

We used two images (that were not included during the training step) to test the performance of each DL model at segmenting individual nuclei. Each DL model with the best performance metrics depicted in Table 3 was compared against the manual annotation (ground-truth).  Figure 2 depicts the performance for each model at different *T* (threshold) values, ranging from 0.1 (low overlap between the predicted nucleus and the manual annotation) to 0.7 (high overlap), for the evaluation metrics detailed in Materials and Methods. Overall, Cellpose was the best performer across different metrics, particularly at greater thresholds (higher overlap stringencies). Stardist and SplineDist had similar performances, which is not surprising, since cell nuclei have a convex shape and SplineDist was designed to outperform Stardist at predicting nonconvex structures. The worst performer was U-Net3-class, lower than even the default pretrained Stardist model. In cases where training cannot be performed, the Stardist (pretrained model) might give acceptable performance; however, training a DL platform with manually annotated images, particularly those specialized for nuclei segmentation, will generate a model with improved performance (compare the blue and red lines in Figure 2).

### 3.5. Comparison of Machine Learning Algorithms and Nuclei Segmentation Models

**Figure 3:**
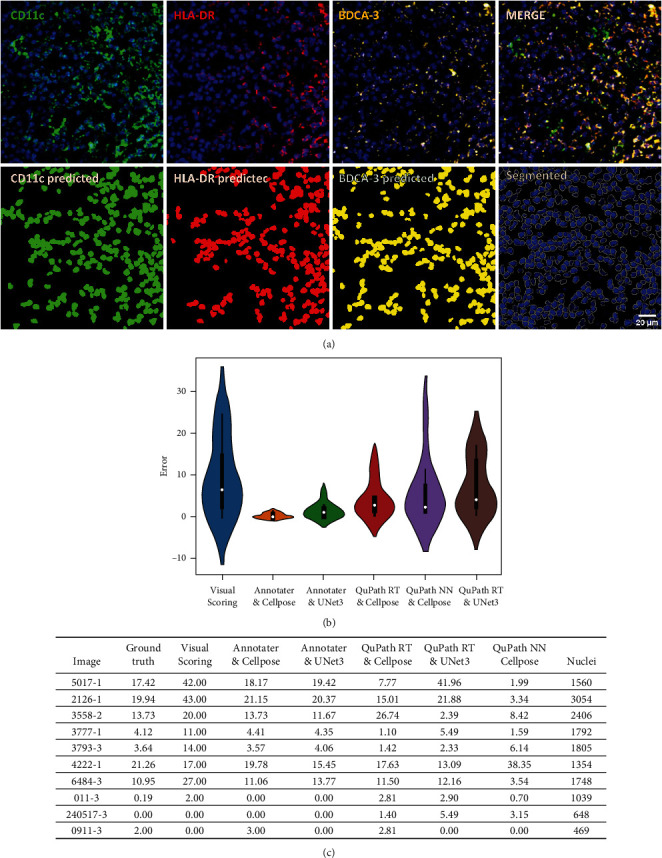
Performance evaluation of machine learning classifiers and nuclei segmentation algorithms: Annotater and Cellpose. (a) Examples of images of the marker prediction with the classifiers trained with logistic regression. The images show the immunofluorescence (IF) staining for cDC1 typical markers CD11c (green), HLA-DR (red), and BDCA-3 (cyan), each shown with the corresponding nuclei stain (blue), plus a merged image of the four channels; below are the mask images of predicted positive cells per marker. (b) Violin graph showing the distance of different ML classifiers to the ground-truth (percentage of cDC1 quantified manually considering the total number of cells in the field). (c) Percentages of cDC1s from the total of nuclei in the three images of high immune infiltration obtained by different trained models. Ground-truth represents the total of cDC1s identified manually in the training images. Visual scoring represents the classical way of quantifying, considering only 100 cells per field. In (b), RT denotes random trees and NN denotes neural networks.

After obtaining the cell nuclei segmentation models and evaluating their performance, we compared different ML algorithms for the task of cell classification using the same cohort of training images used to generate our own models of segmentation. Annotater, an ImageJ plugin, was selected as the first marker annotation tool. This plugin trains ML-based classifiers for each marker using the logistic regression algorithm. We also used QuPath, a software program used in pathology and image analysis that offers a more fluid interface. Two ML algorithms were implemented to perform measurements (artificial neural networks (ANN) and random trees (RT)).  Figure 3(a) shows representative images of cells positive for cDC1 markers and the predicted positivity obtained with the trained classifiers using Annotater, showing at visual level the performance of this tool. To evaluate the relative performance of different ML models, we compared all the predicted results of each model to the manually annotated ground-truth and measured the distance or error to this value (Figures 3(b) and 3(c)). Furthermore, the effect of the quality of the segmentation model upon ML algorithm performance was assessed by applying it to the Cellpose (best quality) and U-Net3-class (worst quality) segmentation model results. We found that cells segmented with Cellpose followed by marker identification with logistic regression in Annotater produced the lowest error (deviation from the ground-truth), whereas, as expected, combining Annotater with the cell segmentation results from the U-Net3-class DL model was less successful. Compared to Annotater, the performance of both the QuPath random trees and the ANN ML models with both Cellpose and U-Net3-class segmented images was less precise, obtaining in some cases almost the same values as the classical visual scoring (which was the method with the highest level of error). Although the use of three fields per patient to evaluate cell counts on visual scoring could reduce this high error rate, this result emphasizes the improvements that are achieved with AI techniques, as well as the importance of evaluating a number of approaches to identify the optimal method for a given dataset.

### 3.6. Evaluation of the Abundance of cDC1s in the Tumor Microenvironment

**Figure 4:**
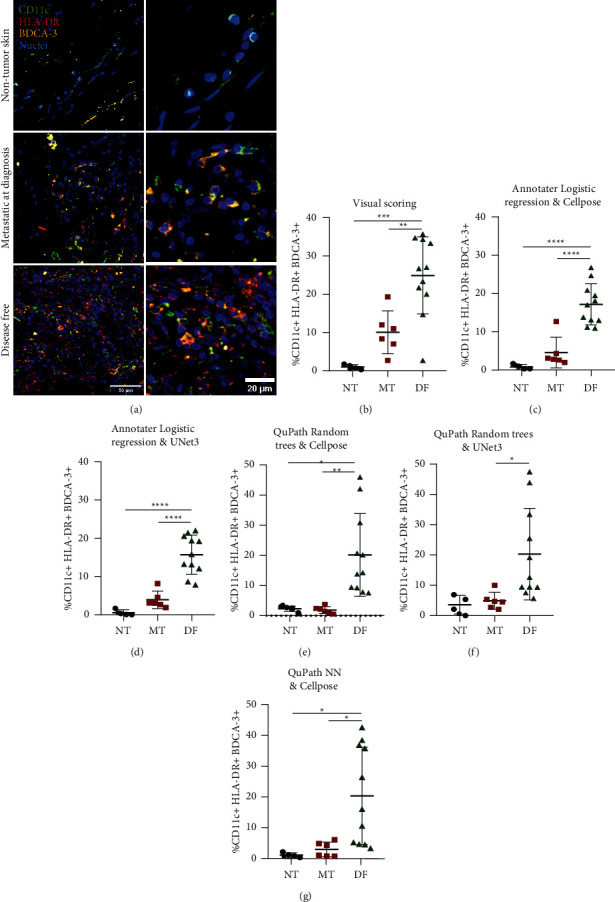
Identification of classical dendritic cells 1 (cDC1s) as an important immune population for tumor control using deep learning-based segmentation and machine learning classifiers. (a) Representative conventional IF images per group of study. The right column represents digital zooms. Percentage of CD11c^+^ HLA-DR^+^ BDCA-3^+^ cDC1s quantified by visual scoring (b), Annotater by logistic regression and using object mask generated by Cellpose (c), Annotater by logistic regression and U-Net3-class (d), QuPath by random trees and Cellpose (e), QuPath by random trees, U-Net3-class (f), and QuPath by artificial neural network and Cellpose (g). Percentages were estimated with respect to all the nucleated cells in the image. These plots included the 54 images used for the final analysis, plots show median ± standard deviation, and statistical data were pooled from patients with metastasis at diagnosis (MT, *n* = 6), disease-free (DF, *n* = 11), and nontumor skin (NT, Ctrl, *n* = 5) using one-way ANOVA with multiparametric comparison. ^*∗*^*p* < 0.05,^*∗∗*^*p* < 0.01,^*∗∗∗*^*p* < 0.001,  and ^*∗∗∗∗*^*p* < 0.0001.

We evaluated the cDC1 abundance in the TME using different DL-ML models. For this, we used all the IF images, excluding the training cohort (54 images total). Each ML classifier (logistic regression in Annotater and ANN and RT in QuPath) was evaluated using the best (Cellpose) and worst (U-Net 3-class) performers of the DL nuclei segmentation. Comparisons were made against the data obtained with visual scoring.  Figure 4(a) shows representative IF images and Figures 4(b)–4(g) show quantitative plots for the nontumor skin of control, metastatic, and disease-free groups. We observed a significantly higher abundance of cDC1s in biopsies of patients controlling the disease than in those metastatic or nontumor biopsies at visual level. This result was consistent between visual scoring and any of the DL-ML models. Although all the strategies reported similar results and suggested a positive correlation between cDC1 abundance and disease control, Annotater yielded results with lower dispersion and higher statistical significance (Figure 4(d)). This confirmed the result obtained in Figure 3, also highlighting the importance of evaluation and selection of ML models with good performance to directly assess the biological significance of an immune population in cancer progression. The abundance of cDC1s was based on the evaluation of areas of high levels of infiltrating cells, but, in order to evaluate whether the distribution was the same across the tumor, we scanned the complete slide of IF staining, also including the tumoral marker HMB45. We confirmed in representative patients that metastatic samples have low density of cDC1 across the tumor compared with disease-free samples (Supplementary Figures 1(a) and 1(b)). This result supports the analysis of particular melanoma regions with a high immune infiltration and the association of cDC1s with disease control.

We concluded that Cellpose was the best DL model to segment nuclei and that together with Annotater it can efficiently identify immune cells in the TME of melanoma. Coupled with this enhanced performance, these two models have a greater user friendliness compared with QuPath and only need basic programming skills (Supplementary Table 2 and Supplementary Video 1). We selected Cellpose and Annotater for subsequent processing and analysis of mIF images. Supplementary Figure 2 and Supplementary Table 2 summarize the workflow used to establish the optimized image-based ML approach for inspection of the immune infiltrate in tumors, taking into account the capabilities of each laboratory.

### 3.7. cDC1s Show a Predominantly Activator Phenotype in Disease-Free and Tolerogenic Profile in Metastatic Patients

**Figure 5:**
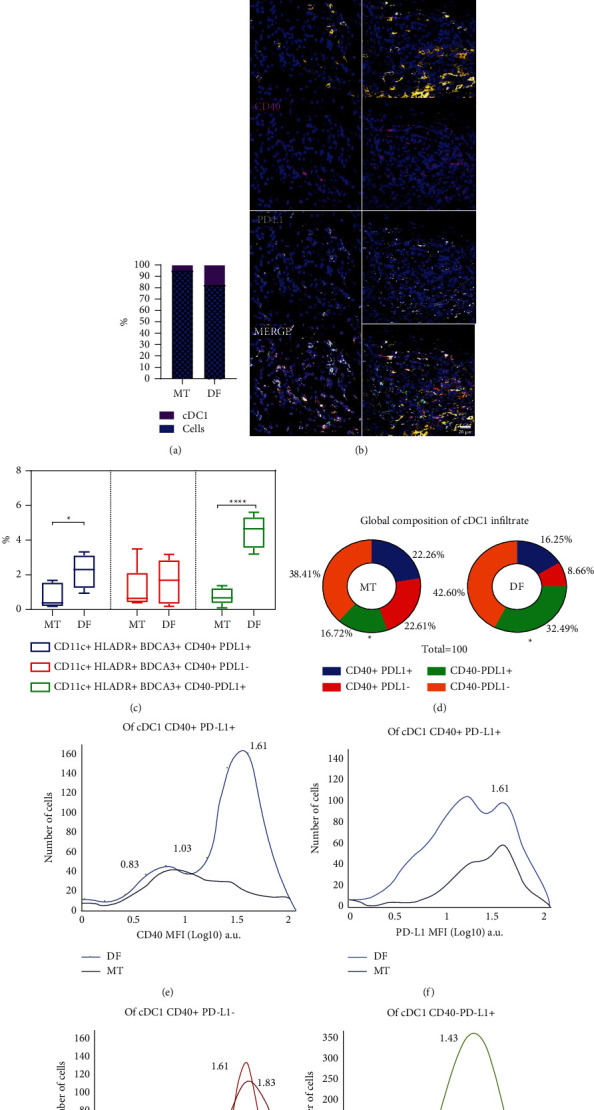
Analysis of CD40 and PD-L1 expressions on cDC1s using the machine learning-based workflow. (a) Abundance of cDC1s in image fields rich in immune infiltrate. (b) Representative multiplexed immunofluorescence micrographs for CD11c (green), HLA-DR (red), BDCA-3 (yellow), CD40 (magenta), PD-L1 (gray), and nuclei (blue) in metastatic (MT, *n* = 5) and disease-free (DF, *n* = 5) patients. (c) Abundance of different cDC1 (CD11c^+^ HLA-DR^+^ BDCA-3^+^) cells expressing CD40, PD-L1, or both markers. Percentages were estimated with respect to all the nucleated cells in the image including three fields per patient; boxes represent the median with the lowest and highest quartiles, the whiskers, and the maximum and minimum values. (d) Pie charts of the cDC1 subpopulations based on expressions of PD-L1 and CD40. ((e)–(h)) Normalized mean fluorescence intensity for CD40 ((e) and (g)) or PD-L1 ((f) and (h)) on double-positive ((e)-(f)), CD40^+^ PD-L1^−^ (g), and CD40^−^ PD-L1^+^ (h) cDC1s. Arbitrary fluorescence units Log10 scale. Statistical data were pooled from patients using Student's *t*-test, ^*∗*^*p* < 0.05, ^*∗∗∗∗*^*p* < 0.001.

Our ML-based workflow allowed us to observe a significantly enhanced infiltration of cDC1s in the TME of melanoma patients that control the disease, supporting this population as critical for the antitumor response. Indeed, we observed that, on average, taking images of tumor fields with an extensive infiltration of immune cells, of all nucleated cells, about 17% are cDC1s in disease-free patients (Figure 5(a)). We then tested the capacity of this ML-based strategy to identify additional cDC1 markers related to a potential activator or inhibitory phenotype on images generated by mIF staining. Five metastatic patients at diagnosis and five disease-free patients were randomly selected to evaluate the expression of the coactivator receptor CD40 and inhibitory receptor PD-L1 (30 images in total) (Figure 5(b)). We found that both CD40^+^ and PD-L1^+^ cDC1s were increased in disease-free patients (Figures 5(c) and 5(d)). Interestingly, we observed a cDC1 population that coexpresses both markers, which is in agreement with transcriptional studies indicating that in some DC subsets both molecules are coexpressed [14–16]. This double-positive cDC1 population was more abundant in disease-free patients, suggesting a role in disease control despite the expression of PD-L1. Also, cDC1s expressing PD-L1 but not CD40 were more abundant in patients controlling melanoma (Figure 5(c)). We compared the abundance of each cDC1 subset in the two groups of patients. Strikingly, we found that the most substantial difference between patients was the predominance of PD-L1^+^CD40^−^ cDC1s in disease-free patients (32.5% versus 16.7%, Figure 5(d)). We took advantage of the ML-based workflow to evaluate the levels of expressions of CD40 and PD-L1 using the mean fluorescence intensity. We noticed that almost all double-positive cells were CD40^high^ in disease-free patients (Figure 5(e)), while in metastatic patients they were CD40^low^. Also, the PD-L1^low^ subset of double-positive cDC1s was more abundant in disease-free patients than in metastatic patients (Figure 5(f)). No significant differences were observed in the levels of expressions of CD40 and PD-L1 in the single positive cDC1s (Figures 5(g) and 5(h)). Altogether these data suggest an activator role for PD-L1^low^CD40^high^double-positive cDC1s in disease-free patients and a more tolerogenic role for PD-L1^high^CD40^low^double-positive cDC1s in patients with metastases.

**Figure 6:**
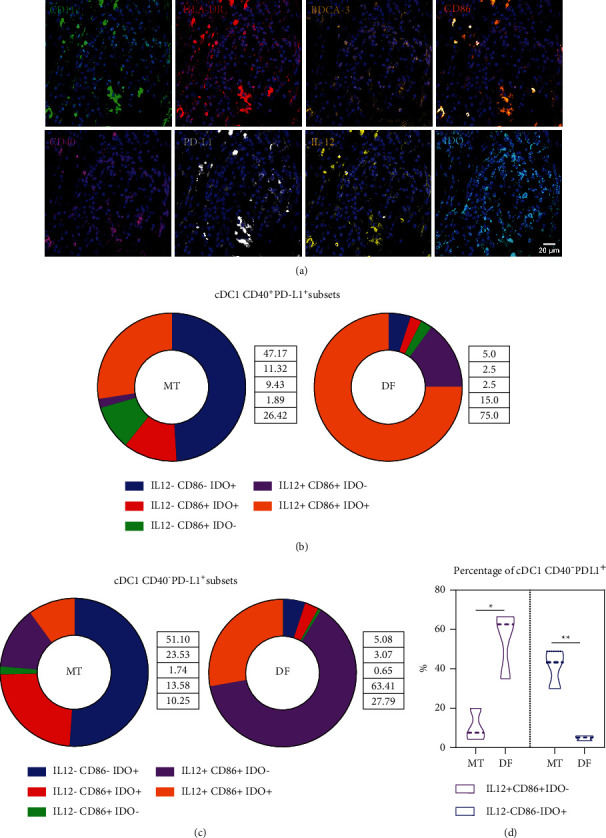
cDC1s expression of IL-12, CD86, and IDO. (a) Representative multiplexed immunofluorescence micrographs for CD11c (green), HLA-DR (red), BDCA-3 (yellow hot), CD86 (orange), CD40 (magenta), IL-12 (yellow), PD-L1 (gray), IDO (cyan), and nuclei (blue) in a disease-free patient. Percentages of cDC1s expressing the markers IL-12^+^ CD86^+^ IDO^−^, IL-12^−^ CD86^+^ IDO^+^, and IL-12^+^ CD86^+^ IDO^+^; in each comparison, 100% was the total of CD40^+^ PD-L1^+^ (b) or CD40^−^ PD-L1^+^ (c) cDC1s. (d) Violin plot showing the percentages of IL-12^+^ CD86^+^ IDO^−^ and IL-12^−^ CD86^−^ IDO^+^ cDC1s that are also CD40^−^ PD-L1^+^ (as in (c)). Statistical data were pooled from metastatic patients at diagnosis (MT; *n* = 3) and disease-free patients (DF; *n* = 3) using Student's *t*-test. ^*∗*^*p* < 0.05,^*∗∗*^*p* < 0.01.

To further explore the activator or inhibitory cDC1 profile, we selected three disease-free and three metastatic patients to extend the analysis (18 images in total). We selected two markers of activation, IL-12 and CD86, and the inhibitory molecule IDO, for a total of nine parameters in the same mIF image (including nuclei, Figure 6(a)). We found that the ML-based workflow was suitable for the analysis of these many markers. When we analyzed the subsets of the CD40^+^ cDC1 cells, we observed an enhanced proportion of cells expressing the activation markers CD86 and IL-12 in disease-free patients (Figure 6(b)). On the contrary, the most abundant subset in metastatic patients was the IL-12^−^ CD86^−^ IDO^+^ cDC1, although there was no statistical significance perhaps because of the small number of CD40^+^ PD-L1^+^ cDC1s (blue column of Figure 5(c)). Strikingly, when we evaluated the CD40^−^ PD-L1^+^ cDC1s, we also observed a significant enrichment of subsets expressing the activation markers without expression of IDO in disease-free patients (Figures 6(c) and 6(d)), while the opposite was observed in metastatic patients, in which the CD86^−^ IL-12^−^ IDO^+^ cDC1 subset was enriched.

Altogether, these data favorably argue about the need to incorporate ML-based tools for the analysis of multiparametric images. Our ML-based workflow allowed us to analyze up to nine different parameters on a viable timescale, also providing evidence that cDC1 cells are more abundant in the TME infiltrate of melanoma patients that control disease. Furthermore, the ML-based workflow also supports that cDC1s in disease-free patients exhibit an expression profile that is more congruent with an activator function: CD40^high^ PD-L1^low^ CD86^+^ IL-12^+^. Although disease-free patients were also enriched of CD40^−^ PD-L1^+^ cDC1s, these cells were also more compatible with an activator phenotype. On the contrary, patients that were metastatic at diagnosis were enriched for cDC1s with a more tolerogenic phenotype (CD40^low^ PD-L1^high^ CD86^−^ IL-12^−^ IDO^+^).

We were surprised to observe a CD40^−^ PD-L1^+^ subset of cDC1 positively correlating with the control of disease. These cells may represent an immature cDC1 or a skin cDC1 that has been activated but has yet to migrate to the lymph nodes, where CD40 expression will be upregulated. Of note, it was recently reported that signaling through an intracellular domain of PD-L1 favors cDC1 migration from the skin to lymph nodes [40], which could explain the high abundance of this cDC1 subset in disease-free patients, also supporting the idea that these cells are of recent activation. In this scenario, CD86 and IL-12 may be better markers than CD40 and PD-L1 to profile melanoma-infiltrating cDC1s with antitumor activity. These data also highlight the importance of a multiparametric analysis, facilitated by the increase in throughput afforded by the use of the most appropriate ML-based model, to obtain a more complete phenotypic profile of the immune cells that infiltrate melanoma and perhaps many other tumors. A better understanding of immune populations that control disease will help to provide the most suitable therapeutic recommendations.

### 3.8. Study Limitations

Implementation of an ML-based workflow allowed us to identify a particular cDC1 profile enriched in disease-free melanoma patients. Despite this, further validation needs to be performed, including larger numbers of patients, samples, and images to refine the analysis and strengthen the workflow. Although this work focuses on evaluating the profile of cDC1, in the future, the relationship of tolerogenic or activating cDC1s with T cells should be assessed. Moreover, it will be relevant to evaluate whether the profiles of cDC1s depend on their localization and distribution in the melanoma tissue, considering that not only the abundance of these cells is important, but also the availability of antigens and their ability to present them to T lymphocytes to promote an effective antitumor response [41, 42]. In this scenario, the spatial distribution of specific cDC1s should also impact on local or distant metastasis, as well as the organs targeted for metastases.

## 4. Conclusions

The analysis of mIF images by ML models amiable to use in a normal laboratory allowed us to find that cDC1s were enriched in melanoma patients that control the disease. Although cDC1s in the melanoma TME exhibited a complex phenotype in which activator and inhibitory molecules were often coexpressed, an activator profile was more consistently observed in disease-free patients, as well as a tolerogenic profile in those that have metastasis. This complex phenotype is in tune with the complexity of signals that immune cells encounter in the TME. To the best of our knowledge, this study is the first that explores different DL-ML tools and combines the use of mIF and AI to profile the expression of nine markers of identity and performance to describe in depth the activator or inhibitory profile of TME-infiltrating cDC1s in clinical samples. The ML-based workflow established in this study allowed us to evaluate a large number of cells in a short period of time and reduce possible subjective operator bias. The use of AI-based automated and semiautomated tools for rapid analysis of large datasets promises to transform the accuracy and speed of identifying cell populations that can provide useful information in a clinical setting.

## Figures and Tables

**Table 1:** Clinical information of the study cohort.

No.	Patient code	Classification	T	N	M	Breslow (mm)	Histologic type	Clinic evolution
1	5532	MT	T4B	1B	0	7	Acral lentiginous	Regional metastases, progression to lung, liver and, mediastinum, and abdominopelvic invasion (local and distant)
2	2847	MT	T4B	NR	IB	12	Nodular	Central nervous system, lung, and liver metastases (distant)
3	4563	MT	T4B	3C	0	13	Acral lentiginous	Lymph node metastases (local)
4	7737	MT	T4B	1	0	11	Spindle cell	Lung and liver metastases (distant)
5	5539	MT	T4B	3C	0	4	Acral lentiginous	Inguinal activity with multiple satellite lesions (local)
6	9337	MT	T4B	1	0	23	Nodular	Regional metastases (local)
7	5406	DF	2	0	0	2	Superficial spreading	Remission
8	2126	DF	1	0	0	0.8	Acral lentiginous	Remission
9	2242	DF	T1A	0	0	0.3	Acral lentiginous	Remission
10	5835	DF	T3	0	0	3	Superficial spreading	Remission
11	1009	DF	2	0	0	1.1	Acral lentiginous	Remission
12	8011	DF	4	1	0	4	Nodular	Remission
13	5017	DF	1	0	0	0.6	Acral lentiginous	Remission
14	6321	DF	TIS	0	0	NA	Acral lentiginous	Remission
15	9242	DF	T4B	0	0	12	Nodular	Remission
16	4953	DF	T3B	0	0	2.2	Nodular	Remission
17	8464	DF	TIS	0	0	NA	Acral lentiginous	Remission

TNM classification: T: tumor, N: node, M: metastases (also MT in classification), NA: no applicable.

**Table 2:** Panels of cDC1 markers stained during the establishment of the workflow and the final analysis.

Immunofluorescence images shown in Figures 1–4			
	CD11c revealed with AF488	HLA-DR revealed with AF694	BDCA-3 revealed with AF647

Immunofluorescence images shown in Figure 5			
Base staining	CD11c revealed with AF488	HLA-DR revealed with AF694	BDCA-3 revealed with AF647
First cycle		Anti-CD40PE-conjugated	Anti-PD-L1 APC-conjugated

Immunofluorescence images shown in Figure 6			
Base staining	IDO revealed with AF488	CD40 revealed with AF494	BDCA-3 revealed with AF647
First cycle	Anti-CD11cFITC-conjugated	Anti-HLA-DR PE-conjugated	Anti-PDL1APC-conjugated
Second cycle		Anti-CD86PE-conjugated	Anti-IL12APC-conjugated

**Table 3:** Optimal parameters settings by selecting the models with the highest *F*1-Score.

Model	Patch size	Epoch	Batch size	Augmentation	Learning rate	Training time
U-Net3-class	1024 × 1024	500	2	5	0.01	2 h 50 min
Stardist	1024 × 1024	250	2	5	0.0003	1 h 44 min
SplineDist	512 × 512	200	4	5	0.0003	32 h 36 min
Cellpose	1024 × 1024	20,000	2	5	0.0002	2 h 37 min

## Data Availability

The data used to support the findings of this study can be obtained from the corresponding author upon request.

## Supplementary Materials

Supplementary Materials Supplementary Table 1: dataset for manual annotation. Supplementary Table 2: comparison between Annotater and QuPath. Supplementary Figure 1: expanded view of melanoma samples showing the distribution of cDC1s. Supplementary Figure 2: a schematic diagram for decision-making to implement a deep learning-based workflow of image analysis. Supplementary Video 1: example of detections by machine learning classifier per each marker with Annotater.
