# Supplementary material for: A Machine Learning Workflow of Multiplexed Immunofluorescence Images to Interrogate Activator and Tolerogenic Profiles of Conventional Type 1 Dendritic Cells Infiltrating Melanomas of Disease-Free and Metastatic Patients
Source: J Oncol. 2022 Oct 12;2022:9775736. doi: 10.1155/2022/9775736 (PMC9581597; doi:10.1155/2022/9775736)
Supplement: Supplementary Materials — Supplementary Table 1: dataset for manual annotation. Supplementary Table 2: comparison between Annotater and QuPath. Supplementary Figure 1: expanded view of melanoma samples showing the distribution of cDC1s. Supplementary Figure 2: a schematic diagram for decision-making to implement a deep learning-based workflow of image analysis. Supplementary Video 1: example of detections by machine learning classifier per each marker with Annotater. [file 9775736.f1.pdf]

Supplementary Materials

Table S1. Data set for Manual Annotation.

Image	Time of annotation	Nuclei annotated
**5017-1**	3 h 40 min	1560
**2242-1**	3 h 04 min	1354
**8464-3**	4 h 15 min	1748
**6090-1**	5 h 30 min	2184
**1628-1**	6 h 05 min	2501
**1134-2**	4 h 10 min	1774
**5835-2**	5 h 50 min	2406
**7737-1**	5 h 12 min	1792
**9337-3**	5 h 00 min	1805
**2405-3**	1 h 17 min	648
**0101-3**	2 h 00 min	1039
**0900-1**	1 h 00 min	469

Table S2. Comparison between Annotater and QuPath

	Advantages	Disadvantages
**Annotater**	Integrated with ImageJ.<br>Do not need programming skills.<br>Training of classifiers is intuitive.<br>Obtaining the results is an integrated function.	The time spent in the batch analysis is longer than QuPath.
**QuPath**	Automated analysis is easier to implement.<br>The time spent in the batch analysis is shorter than the Annotator.<br>Optimal visualization of markers.<br>There are different options to normalize the data.	Need programming scripting skills.<br>Only StarDist could be integrated to segment nuclei.<br>Different masks of segmented nuclei requires different treatments<br>Training of classifiers is less intuitive than Annotator

a

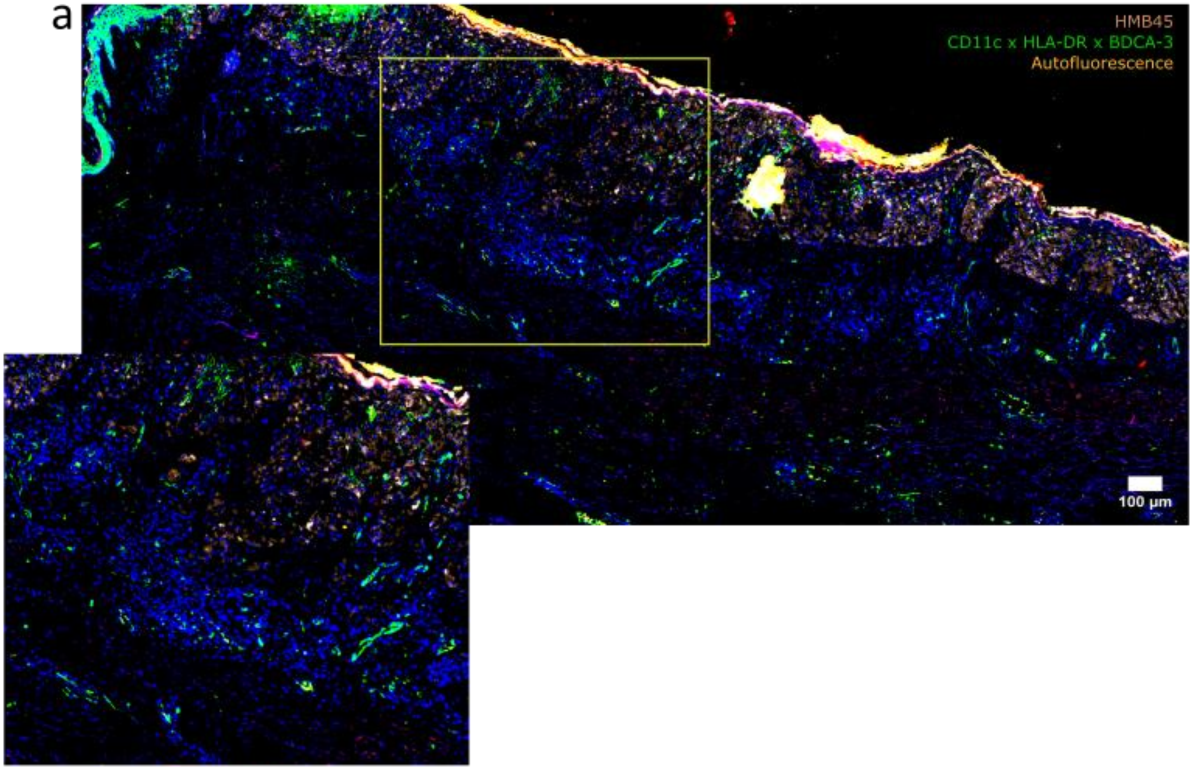
HMB45
CD11c x HLA-DR x BDCA-3
Autofluorescence
100 µm

b

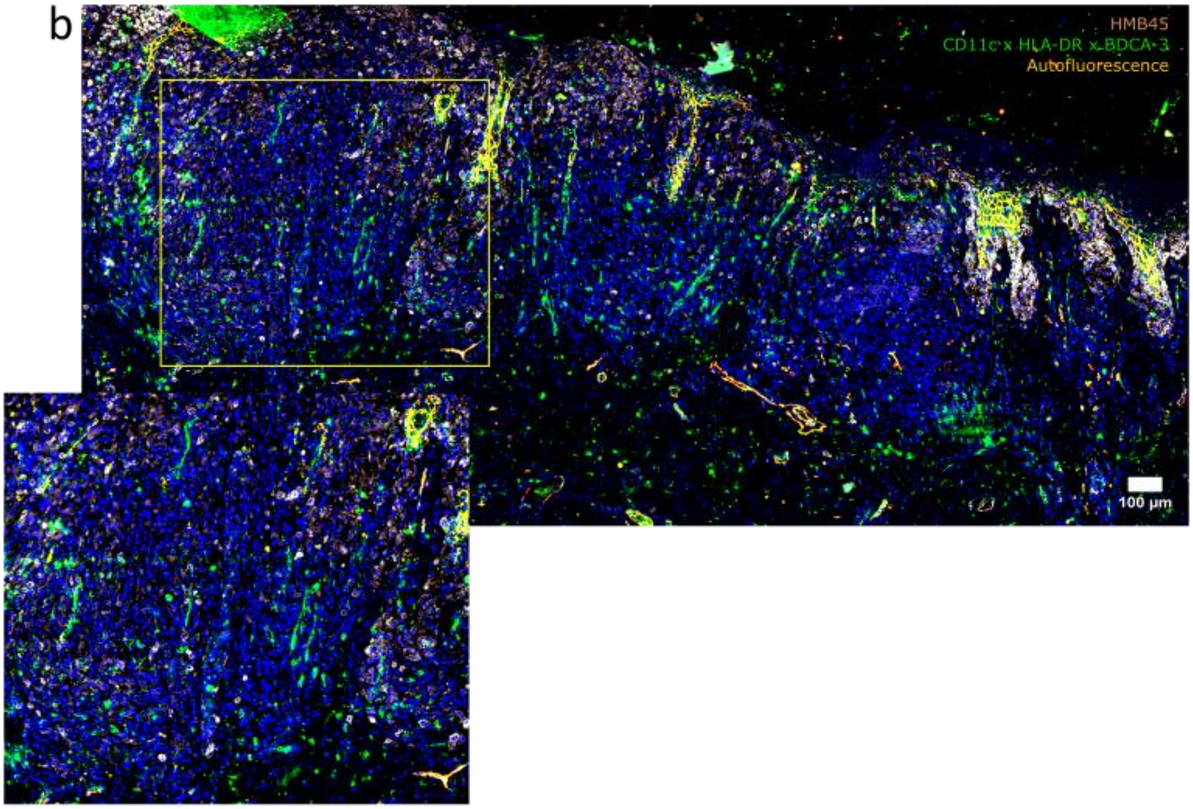
HMB45
CD11c x HLA-DR x BDCA-3
Autofluorescence
100 µm

Supplementary figure 1. Expanded view of melanoma samples showing the distribution of cDC1s. Slide scanning of an expanded region of melanomas from a patient (a) metastatic at diagnosis and (b) disease free showing the distribution of cDC1 (green) respect to the tumor cell (HMB45pos-sepia). cDC1 were identified using CD11c, HLA-DR and BDCA3, these signals were treated mathematically to show only the areas of coincidence in green. The squares in yellow are areas magnified (below).

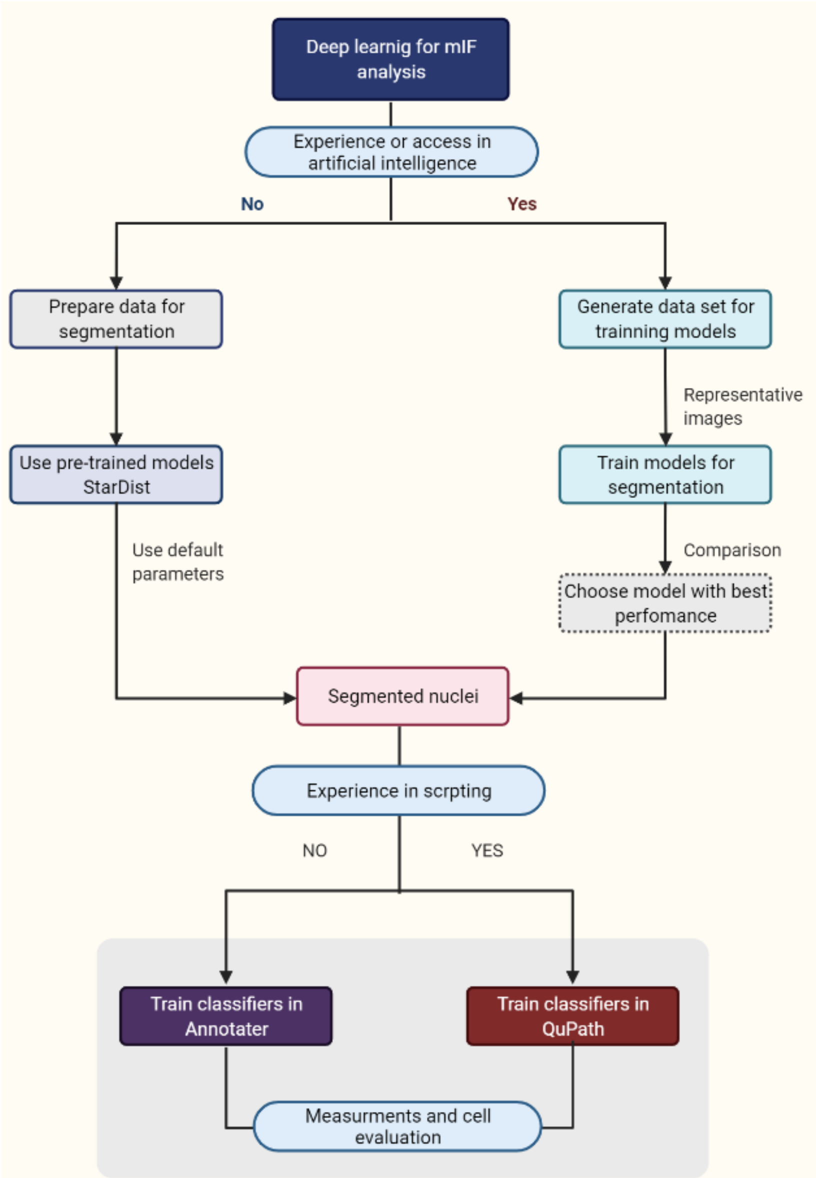

Supplementary Figure 2. A schematic diagram for decision-making to implement a deep learning-based workflow of image analysis.

Supplementary Video 1. Example of detections by machine learning classifier per each marker with Annotater.

https://drive.google.com/file/d/19jY3PnVd36EuP_Qdc1_Eb0ifyPQcmul9/view?usp=sharing
